# A CREB2-targeting microRNA is required for long-term memory after single-trial learning

**DOI:** 10.1038/s41598-018-22278-w

**Published:** 2018-03-02

**Authors:** Sergei A. Korneev, Dimitris V. Vavoulis, Souvik Naskar, Varvara E. Dyakonova, Ildikó Kemenes, György Kemenes

**Affiliations:** 1 Sussex Neuroscience, School of Life Sciences, University of Sussex, Brighton, BN1 9QG UK; 2 RDM Nuffield Division of Clinical Laboratory Sciences, University of Oxford, Clifton, BS8 1UB UK; 3 Koltzov Institute of Developmental Biology, Russian Academy of Sciences, Moscow, 119334 Russia

## Abstract

Although single-trial induced long-term memories (LTM) have been of major interest in neuroscience, how LTM can form after a single episode of learning remains largely unknown. We hypothesized that the removal of molecular inhibitory constraints by microRNAs (miRNAs) plays an important role in this process. To test this hypothesis, first we constructed small non-coding RNA (sncRNA) cDNA libraries from the CNS of *Lymnaea stagnalis* subjected to a single conditioning trial. Then, by next generation sequencing of these libraries, we identified a specific pool of miRNAs regulated by training. Of these miRNAs, we focussed on Lym-miR-137 whose seed region shows perfect complementarity to a target sequence in the 3’ UTR of the mRNA for CREB2, a well-known memory repressor. We found that Lym-miR-137 was transiently up-regulated 1 h after single-trial conditioning, preceding a down-regulation of *Lym-CREB2* mRNA. Furthermore, we discovered that Lym-miR-137 is co-expressed with *Lym-CREB2* mRNA in an identified neuron with an established role in LTM. Finally, using an *in vivo* loss-of-function approach we demonstrated that Lym-miR-137 is required for single-trial induced LTM.

## Introduction

Our present understanding of small non-coding RNAs (sncRNAs) began with the pioneering work of Lee *et al*. who discovered that the *lin-14* gene in *C*.*elegans* is regulated by two short RNAs, *lin-4S* and *lin-4L*^1^. Since then there have been an enormous number of advances in our knowledge about the diversity and potential functions of sncRNAs. Particularly significant progress has been made in studies of microRNAs (miRNAs), which are 20–23 nucleotides in length and mostly act as negative regulators of gene expression. The reported prevalence of miRNAs in the CNS suggests their important role in brain function [for a review, see^2^]. Although recent studies already have implicated miRNAs in long-term synaptic plasticity and memory in both invertebrate and vertebrate models of learning and memory^3–7^, it is unknown whether specific miRNAs play key roles in the consolidation of associative LTM after single-trial classical conditioning and if yes, what their downstream targets are. This is an important question to address because although single-trial induced long term memories, including ‘flashbulb’ memories in humans, have been of major interest in neuroscience^8^, it is still unclear how a single episode of learning can activate the complex molecular machinery normally required for the formation of LTM. Here we test the hypothesis that miRNAs, particularly those with an ability to downregulate the expression of known inhibitory constraints of memory consolidation, are an important component of this process.

A particularly favourable model to study the role of non-coding RNAs in memory formation after single-trial classical conditioning is provided by the mollusc *Lymnaea stagnalis*^9–11^. In this species, a single pairing of amyl acetate, a neutral chemical conditioned stimulus (CS) and sucrose, an unconditioned feeding stimulus (US) leads to LTM^12,13^. A unique advantage of this system is that the links between learning-induced molecular changes and LTM can be followed in a precisely timed manner relative to the time of acquisition.

By using pharmacological blockade of Dicer, a ribonuclease of critical importance for miRNA biogenesis, we first established that the miRNA pathway in general is required for single-trial induced LTM formation in *Lymnaea*. We then constructed and sequenced sncRNA cDNA libraries prepared from the ‘learning’ ganglia, the buccal and cerebral ganglia, in which the memory trace is formed, stored and expressed after food-reward conditioning^14,15^. We identified a limited pool of miRNAs, the expression of which was differentially regulated by single-trial training. We then concentrated on a specific miRNA, Lym-miR-137, which was transiently up-regulated at 1 h after conditioning. miR-137 has been recently implicated in brain disorders^16^ but few studies exploring the possible role of miR-137 in memory have been conducted and they generated some controversy^17,18^. Here we show that Lym-miR-137 targets mRNA encoding cAMP-response element binding protein 2 (CREB2), an important transcription factor, which, along with its mammalian ortholog, ATF4, is implicated in memory repression^19–22^. Next, we demonstrate that the transient training-induced up-regulation of Lym-miR-137 is followed by down-regulation of *Lym-CREB2* mRNA. Finally, we employed an *in vivo* loss-of-function approach and discovered that the injection of a specific Lym-miR-137 inhibitor before single-trial training increased the level of *Lym-CREB2* mRNA and suppressed LTM in the same conditioned animals. Taken together our results suggest that Lym-miR-137 is required for single-trial induced LTM formation via down-regulation of *Lym-CREB2* mRNA expression in neurons of the learning circuit.

## Results

### Blockade of Dicer activity impairs single-trial induced LTM

**Figure 1:**
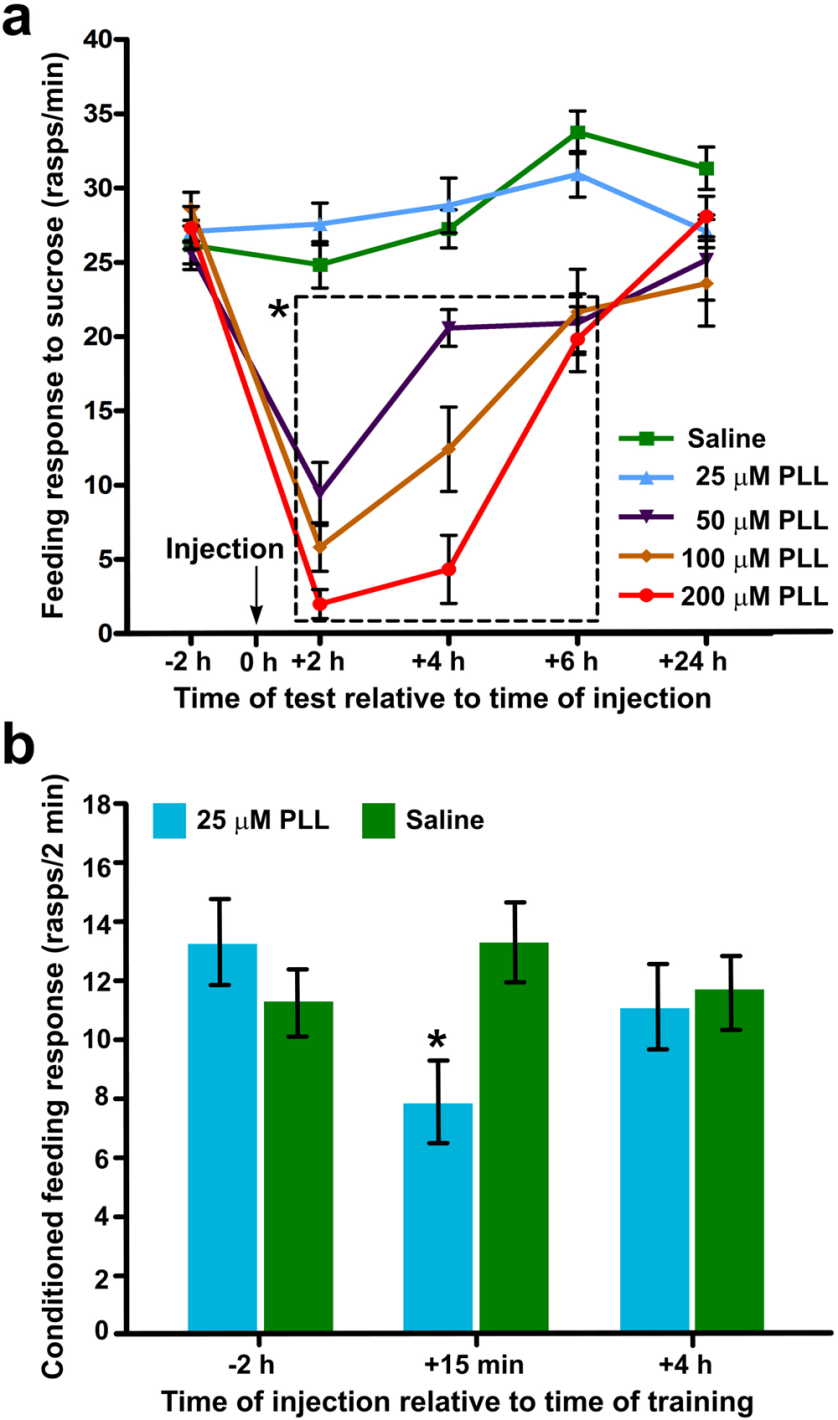
Inhibition of Dicer leads to the impairment of LTM formation. (**a**) Optimization of Poly-L-Lysine (PLL) concentration. The results of sucrose test conducted on snails injected with saline solutions containing PLL at different concentrations (0 µM, 25 µM, 50 µM, 100 µM or 200 µM). The tests were performed 2 h before (−2 h) and 2 h (+2 h), 4 h (+4 h), 6 h (+6 h) and 24 h (+24 h) after the injection. The 50 µM, 100 µM and 200 µM PLL solutions all cause significant impairments of the feeding response at 2 h, 4 h and 6 h post-injection compared against saline treatment (Two-way Repeated Measures ANOVA, p < 0.0001 for both Time and Treatment); Bonferroni posttests, p < 0.001 to 0.05 (compared against saline treatment at each of the post-injection test time points). By contrast, 25 µM PLL does not inhibit the unconditioned feeding response of snails at any of the test time points (Bonferroni posttests, p > 0.05 at each test time point). The asterisk next to the dashed rectangle indicates that all values within the rectangle are significantly lower than the equivalent values in the Saline as well as the 25 µM PLL group. (**b**) The effect of PLL injection on memory formation was studied in 3 experimental (blue bars) and 3 control groups (green bars). All snails were subjected to one-trial CS + US training. 2 h before (−2 h) or 15 min (+15 min) and 4 h (+4 h) after the training, experimental animals were injected with 25 µM PLL, whereas control animals were injected with saline only. All snails were tested for feeding response to the CS (conditioned response) 24 h after the training. An unpaired t-test revealed a significant suppression of the conditioned response (p < 0.02) of experimental animals injected with PLL 15 min after conditioning compared to the control snails. All data in this figure are shown as means ± SEM.

We used Poly-L-Lysine (PLL) to assess the general importance of miRNAs in the formation of associative LTM in *Lymnaea*. PLL is a well-known inhibitor of Dicer^23^, a double-stranded RNA endoribonuclease playing a central role in miRNA biogenesis. We hypothesized that if PLL was found to affect the consolidation of LTM after single-trial classical conditioning, it would indicate a role for the miRNA pathway in this process. First, we examined the potential adverse effects of PLL on the unconditioned feeding behavior of snails. Four groups of animals injected with either 25 µM or 50 µM or 100 µM or 200 µM PLL were subjected to a sucrose feeding test^24^ at different time points before and after systemic injection of the animals with PLL. Statistical comparisons of the behavioral data by Two-way Repeated-measures ANOVA followed by Bonferroni posttests revealed that compared to a saline-injected control group, the feeding responses of the 50 µM, 100 µM and 200 µM PLL-injected groups were significantly suppressed at 2 h, 4 h and 6 h after injection (Fig. 1a, see figure legend for detailed test statistics). This result indicated that PLL at these concentrations exerts obvious adverse effects on the feeding behavior during the period of memory consolidation and therefore could not be used in our further studies. By contrast, there was no significant suppression of the feeding response to sucrose attributable to the injection of 25 µM PLL at any of the time points examined. Based on this result, we selected 25 µM PLL for the miRNA silencing experiments. Three experimental and three control groups of animals were used in these studies. Snails from the experimental groups were injected with 25 µM PLL either 2 h before training or 15 min or 4 h after training, whereas snails from the control groups were injected with saline only. To investigate the effect of the injections on LTM formation, all animals were presented with the CS 24 h after training. We observed a significant suppression of the conditioned feeding response of experimental animals injected with PLL 15 min after conditioning compared to the control snails (Fig. 1b). There was no significant suppression attributable to the injection of PLL performed 2 h before or 4 h after training. This finding allowed us to conclude that the time period during which mature miRNAs are required for the process of memory consolidation was likely to start shortly after training and end within a few hours.

### Next generation sequencing reveals miRNAs differentially regulated by training

**Figure 2:**
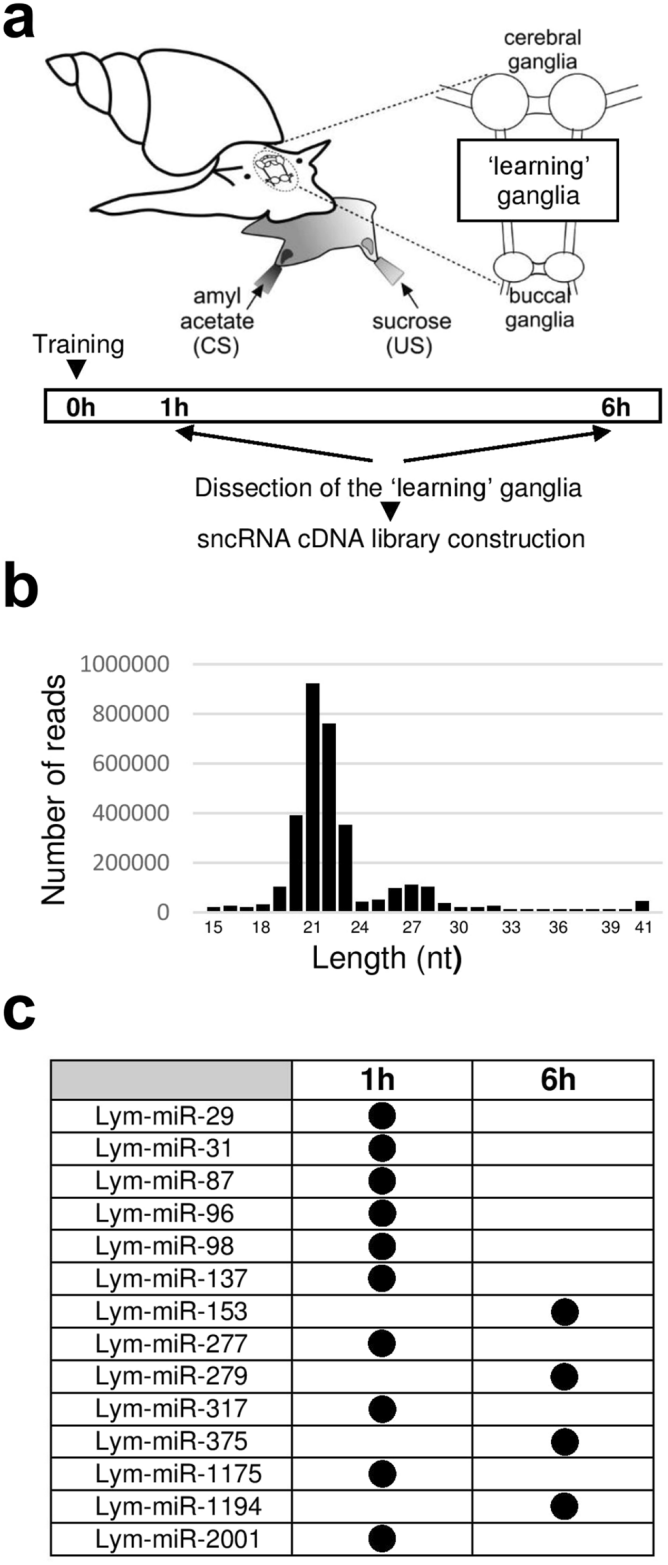
Next generation sequencing reveals a set of miRNAs differentially regulated by single-trial classical conditioning. (**a**) Schematic representation of the experiment to investigate whether single-trial reward conditioning is associated with timed changes in the expression of miRNAs in the ‘learning’ ganglia. (**b**) A size histogram of reads produced by next-generation sequencing of sncRNA cDNA library constructed from the ‘learning’ ganglia extracted from naïve animals. Two main classes of sncRNAs are present: 20–23 nt (mature miRNAs) and 26–28 nt (Piwi-interacting RNAs). Please note that there is also a minor 41-nt class of sncRNAs. (**c**) Differentially expressed miRNAs.

In order to reveal specific miRNAs with a potential role in the consolidation of LTM in *Lymnaea*, we constructed sncRNA cDNA libraries from the ‘learning’ ganglia extracted from conditioned animals at 1 h and 6 h after training (Fig. 2a) as well as from naïve control snails. The 1 h time point was chosen because it is well within the period of general miRNA dependence of LTM revealed in the PLL experiments (between 15 min and 4 h post-training), whereas the 6 h time point was chosen because it is well outside it. We reasoned that this would help identify specific miRNAs that only affect memory consolidation in the period of its general miRNA dependence identified in the PLL experiments.

The sncRNA cDNA libraries were subjected to next generation sequencing. All reads in the libraries were of excellent quality (per-base quality score was >20 for all bases in all reads) and the same two main groups of sncRNAs were identified in all three libraries: 20–23 nt and 26–28 nt (Fig. 2b). The observed distribution of sncRNAs in the libraries was generally in line with the results of previous studies conducted on *Aplysia*^25^. The 20–23 nt group is apparently represented by classical mature miRNAs, whereas the 26–28 nt group is likely to be comprised of piwi-interacting sncRNAs (piRNAs).

The reads were then mapped to the miRBase. Approximately 74% of the reads in the ‘naïve’ and ‘6 h’ libraries and 69% of the reads in the ‘1 h’ library corresponded to known miRNAs. We have compared the abundance of reads corresponding to known individual miRNAs between the libraries we constructed. This analysis resulted in the discovery of 14 miRNAs differentially regulated by single-trial classical conditioning (Fig. 2c).

### Computational analysis indicates that Lym-miR-137 targets *Lym-CREB2* mRNA

**Figure 3:**
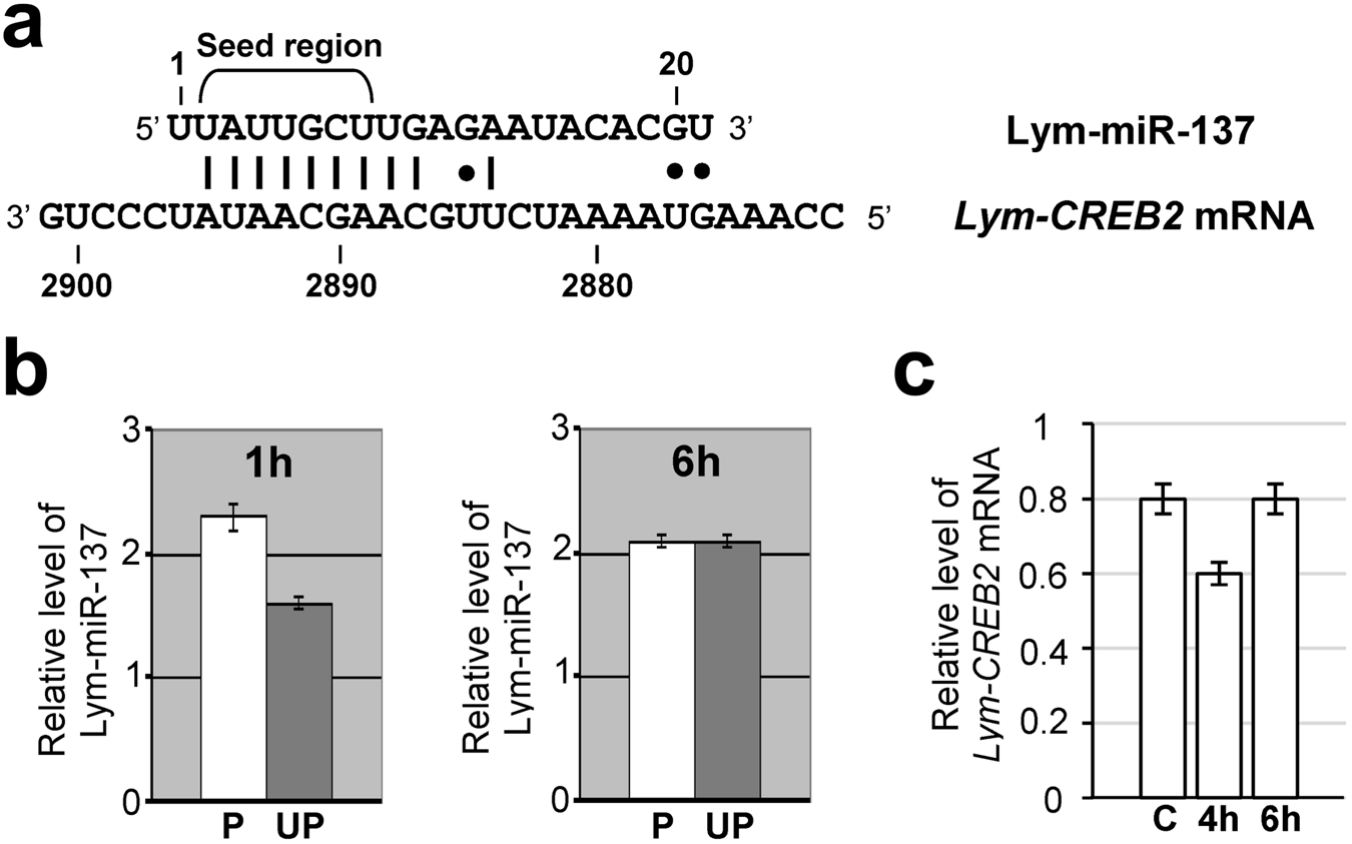
Lym-miR-137 targets *Lym-CREB2* mRNA. (**a**) Alignment of *Lymnaea* CREB2-encoding mRNA (GenBank accession number, AB083656) with Lym-miR-137. Note that the seed region of the Lym-miR-137 exhibits 100% complementarity to the target site located within the 3’ UTR of the *Lym-CREB2* mRNA. The non-Watson–Crick G–U base pairs are shown by dots. (**b**) Quantitative RT-PCR conducted on the ‘learning’ ganglia dissected 1 h and 6 h after training from conditioned (paired, P, n = 10 sets of ‘learning ganglia’ per time point) and control (unpaired, UP, n = 10 sets of ‘learning ganglia’ per time point) animals shows up-regulation of Lym-miR-137 at 1 h but not 6 h after training. The level of Lym-miR-137 expression normalized to an endogenous control (Lym-miR-315) and relative to a calibrator is shown. (**c**) Quantitative RT-PCR conducted on the cerebral ganglia (n = 10 pairs per group) dissected from conditioned (4 h and 6 h after training) and naïve control (C) animals shows down-regulation of *Lym-CREB2* mRNA at 4 h after training. The level of *Lym-CREB2* mRNA expression normalized to an endogenous control (β-tubulin) and relative to a calibrator is shown. All data in this figure are shown as means ± SEM.

The task of finding potential RNA targets for the identified differentially expressed miRNAs was achieved through computational analysis of available molluscan RNA data. Notably, the analysis revealed that the seed region of Lym-miR-137 is 100% complementary to the potential binding site located within the 3’ UTR of *Lym-CREB2* mRNA (Fig. 3a). This suggests that Lym-miR-137 is likely to interact with *Lym-CREB2* mRNA through base-pairing. These findings are intriguing because the CREB2 protein (known as ATF4 in mammals), is an important transcription factor implicated in memory repression in both vertebrates^26^ and invertebrates, including *Drosophila*^20^, *Aplysia*^22,27^ and *Lymnaea*^21,28^. To provide further support for our hypothesis, we then used PITA algorithm^29^ to calculate free energy of the Lym-miR-137/*Lym-CREB2* mRNA duplex formation (ΔGduplex) and total free energy difference (ΔΔG = ΔGduplex − ΔGopen). They were −13.5 kcal/mol and −9 kcal/mol respectively indicating a high binding affinity between Lym-miR-137 and *Lym-CREB2* mRNA.

### Upregulation of Lym-miR-137 after single-trial training is followed by downregulation of *Lym-CREB2*

Data obtained from next generation sequencing indicated that Lym-miR-137 is up-regulated at 1 h after training. Specifically, our calculations showed that the Lym-miR-137 expression level in conditioned animals was approximately 1.5 times higher than in naïve snails at that particular point in time. To verify this result, we conducted real-time RT-PCR on the ‘learning’ ganglia dissected from conditioned and control snails at 1 h and 6 h after the training/control trial. This time we used the un-paired control group of animals to rule out non-associative effects and strengthen the validity of our findings. Notably, these experiments revealed that in classically conditioned animals Lym-miR-137 was indeed up-regulated (approximately 1.5 times) at 1 h after conditioning compared to control animals (Fig. 3b). At the same time, we found no changes in the expression level of Lym-miR-137 between the conditioned and un-paired control groups at 6 h after the training/control trial (Fig. 3b). This result matches perfectly the conclusions of our next generation sequencing experiments. Thus, real-time RT-PCR has confirmed the early training-induced change in the expression of Lym-miR-137.

Our next task was to examine the effect of conditioning on the expression of *Lym-CREB2* mRNA. It is worth mentioning that earlier experiments demonstrated that long term facilitation in *Aplysia* is associated with down-regulation of *CREB2* mRNA in the presynaptic neurons^22^ and that overexpression of CREB2 in these neurons causes decreases in synapse strength^30^. With this in mind, in our studies, we decided to target the cerebral ganglia, which contain the cell bodies of modulatory neurons monosynaptically connected to Central Pattern Generator (CPG) and motor neurons of the feeding circuit located in the buccal ganglia^31^. The most prominent of these presynaptic neurons are the Cerebral Giant Cells (CGCs), which both have a well-established role in LTM in *Lymnaea*^14^ and co-express Lym-miR-137 and *Lym-CREB2* (Fig. S1).

Previous studies have shown that miRNAs predominantly act to decrease target mRNA levels and that this effect is detected several hours after the initial changes in miRNA expression^32^. Consequently, we opted for a 4 h and a 6 h post-training test time to investigate if the observed up-regulation of Lym-miR-137 at 1 h post-training is associated with appropriate alterations in *Lym-CREB2* mRNA expression. Notably, and in line with the results of experiments conducted on *Aplysia*, our quantitative analysis performed on the cerebral ganglia revealed transient down-regulation of the expression of *Lym-CREB2* mRNA in the 4 h experimental group (Fig. 3c). This finding indicates that the regulation of *CREB2* mRNA in the cerebral ganglia, which contains key presynaptic neurons of the learning circuitry, is sensitive to the effects of training and that this training-induced transient down-regulation of the expression of the *CREB2* gene follows the upregulation of Lym-miR-137.

### Twenty-four-hour long-term memory has a late as well as an early requirement for transcription

**Figure 4:**
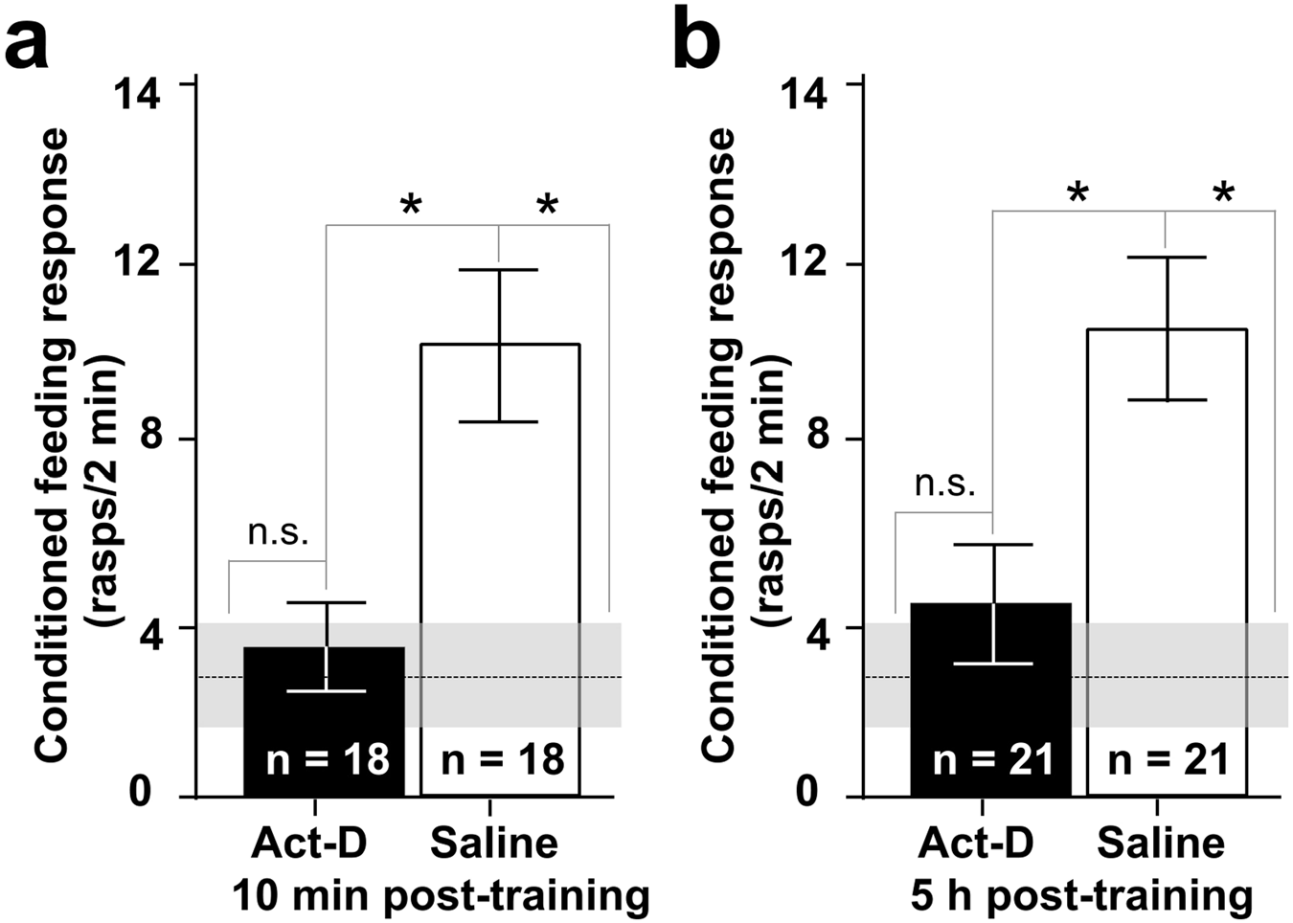
LTM at 24 h is dependent on transcription in both the early and a later stage of memory consolidation after single-trial learning. (**a**) Treatment with Act-D at 10 min post-training reveals that transcription is required for 24 h LTM during the early stage of memory consolidation, around 1 h after single-trial classical conditioning. (**b**) Treatment with Act-D at 5 h post-training reveals that transcription is also required for LTM during a later stage of memory consolidation, around 6 h after single-trial classical conditioning. Means and standard errors are shown for the drug and saline treated groups. The mean non-treated naive baseline level of the feeding response to amyl acetate (n = 20 animals) is indicated by the dashed line with the gray band showing the standard error. Asterisks indicate significant differences revealed by multiple post-hoc tests. Test statistics for data in a: One-way ANOVA, p < 0.001; Tukey’s tests, Saline versus Act-D and baseline, both p < 0.01, Act-D versus baseline, p > 0.05 (not significant, n.s.). Test statistics for data in b: One-way ANOVA, p < 0.0007; Tukey’s tests, Saline versus Act-D and baseline, both p < 0.01, Act-D versus baseline, p > 0.05 (n.s.).

Single-trial learning in *Lymnaea* was originally thought to have a single early time window of transcription dependence^13^ raising the question of the role of the down-regulation of *Lym-CREB2* mRNA at 4 h post-training, observed in the present study. However, the previous study, which demonstrated that transcription is required only for at most the first hour after training, used a 5 h post-training memory test. By contrast, in our current study we used a 24 h post-training memory test because recently there has been substantial progress in the understanding of the molecular mechanisms involved in 24 h LTM^33–36^. Furthermore, these novel data suggested that during the consolidation of 24-h LTM there may be a late as well as an early time-window of transcriptional dependence, underpinning the importance of the down-regulation of CREB2 expression prior to the later time window. Here we directly tested the possible late as well as early transcription dependence of the 24 h LTM by treating animals with the transcriptional blocker actinomycin-D (Act-D). We found that blocking transcription at either 1 h or 6 h post-training (achieved by systemic Act-D treatment at 10 min or 5 h after training) significantly impaired 24-h LTM (Fig. 4).

### Lym-miR-137 is required for LTM formation

**Figure 5:**
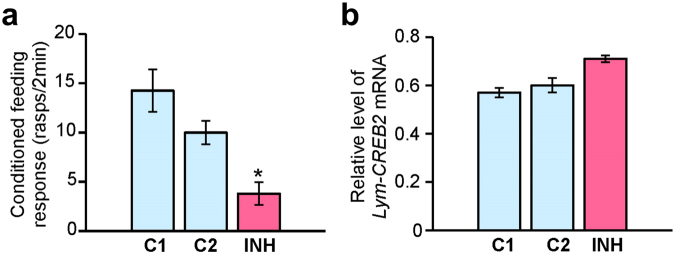
Lym-miR-137 is required for LTM formation. (**a**) Blocking of Lym-miR-137 impairs LTM after single trial conditioning *in vivo* (ANOVA, p < 0.001; Tukey’s tests, INH versus C1 and C2, p < 0.05). Animals (n = 20 per group) were trained 48 h after injection with either the Ls-miR-137 inhibitor (INH) or Invivofectamine only (C1) or miRNA inhibitor negative control (C2). All snails were tested for feeding response to the CS (conditioned response) at 24 h after training. (**b**) Real-time RT-PCR shows that blocking of Lym-miR-137 (INH) causes up-regulation of *Lym-CREB2* mRNA (n = 20 sets of ‘learning ganglia’ per group). All data in this figure are shown as means ± SEM.

The PLL experiments lent strong support to the notion that the miRNA pathway in general is necessary for 24-h LTM in *Lymnaea*. We also identified Lym-miR-137 as a miRNA that is upregulated by single-trial training and may play an important role in the consolidation of memory through the regulation of CREB2, a key molecular constraint on LTM. Here, to test the hypothesis that Lym-miR137 is actually required for 24-h LTM, we employed an *in vivo* loss-of-function approach. In order to down-regulate Lym-miR-137, we used a mirVANA miRNA inhibitor, which produces a highly specific and long-lasting suppressive effect on the target miRNA. However, the miRNA inhibitor-mediated suppression is slow-developing. It is known to take a considerable amount of time (more than 24 hours) before the *in vivo* effect becomes detectable^37^. And indeed, in a first experiment where we used a 24 h pre-training treatment time point no memory impairing effect caused by the application of the Lym-miR-137 inhibitor was revealed (Fig. S3). In a second experiment, we therefore applied a 48 h pre-training treatment with the mirVANA miRNA inhibitor. We made a direct comparison between the conditioned feeding responses of animals injected with the Lym-miR-137 inhibitor, the unrelated negative control inhibitor and Invivofectamine reagent only. Importantly, we found that only the injection of Lym-miR-137 inhibitor 48 h before training had a significant suppressive effect on LTM formation (Fig. 5a). Furthermore, right after the behavioural test, all animals were sacrificed and their ‘learning’ ganglia were subjected to quantitative RT-PCR analysis. Remarkably, we found that *Lym-CREB2* mRNA was up-regulated in the group of snails injected with the Lym-miR-137 inhibitor that showed LTM impairment (Fig. 5b). Thus, our data strongly support the notion that Lym-miR-137 is required for single-trial induced LTM formation via down-regulation of *Lym-CREB2* mRNA expression.

## Discussion

At the heart of the current study is the question of what role miRNAs play in LTM formation after single-trial learning. To begin with, we took a pharmacological approach and showed that inhibition of Dicer shortly after training impaired LTM in *Lymnaea*. This important observation has implications on two fronts. First, it demonstrates that the miRNA pathway is required for single-trial induced LTM formation and suggests that this requirement is restricted to a relatively early time window (less than 4 hours) after single-trial conditioning. Second, it indicates that miRNAs mediate this behavior more by silencing memory repressor genes than by affecting memory enhancer genes. It must be pointed out that these conclusions were somewhat unexpected. Indeed, previous experiments on mice showed that miRNA loss enhanced some types of learning and memory^38^. We believe this discrepancy can be explained by supposing that different learning paradigms are associated with distinct miRNA signatures. Besides, it has to be noted, that PLL might also have been blocking the biogenesis of miRNAs with inhibitory roles in learning and memory (e.g., the *Lymnaea* equivalents of *Aplysia* miR-124^6^ and miR-22^39^). Indeed, the apparent memory impairment achieved by PLL treatment at 15 min post-training was only partial, which could have been due to the competing effects of removing memory-constraining as well as memory-promoting miRNAs. That this is a likely scenario is further supported by the fact that the use of the specific miR-137 blocker resulted in a greater impairment of the conditioned feeding response compared to PLL (compare Figs 1a and 5a).

To identify specific miRNAs with a potential role in LTM we constructed and sequenced sncRNA cDNA libraries from the CNS extracted from conditioned as well as naïve animals. A key outcome of our sequencing data analysis is the discovery of a limited pool of miRNAs differentially regulated by single-trial conditioning. Notably, the revealed training-induced alterations in the expression of miRNAs occurred mainly at 1 h after conditioning indicating an important role of miRNAs during early stages of associative memory formation. Nevertheless, it is also of interest that there is another smaller pool of miRNAs whose expression is altered at 6 h after training suggesting the possible involvement of a diverse pool of miRNAs in the repression of distinct transcripts expressed at different time points after training.

Our search for potential RNA targets for the revealed differentially expressed miRNAs has culminated in the discovery that Lym-miR-137, the expression of which is transiently up-regulated 1 h after training, is likely to interact with mRNA encoding CREB2 protein, an important transcription factor implicated in memory repression. We have provided five lines of evidence in favour of the view that Lym-miR-137 targets *Lym-CREB2* mRNA. First, the ‘seed’ region of the Lym-miR-137 exhibits 100% complementarity to the putative target site located within the 3′ UTR of the *Lym-CREB2* mRNA. Second, the calculated free energy of the duplex formation and total free energy difference indicate a high binding affinity between Lym-miR-137 and *Lym-CREB2* mRNA. Third, learning-induced transient up-regulation of Lym-miR-137 is followed by transient down-regulation of *Lym-CREB2*. Fourth, Lym-miR-137 and *Lym-CREB2* mRNA are co-expressed in an identified neuron with an established role in LTM. Fifth, the injection of miR-137 inhibitor 48 h before single-trial training both increased the level of *Lym-CREB2* mRNA and suppressed LTM in the same animals. Based on the above evidence, we can conclude that in the *Lymnaea* learning and memory circuit Lym-miR-137 plays an essential role in single-trial induced LTM by down-regulating *Lym-CREB2* mRNA expression and thus removing an important molecular constraint of the formation of LTM.

**Figure 6:**
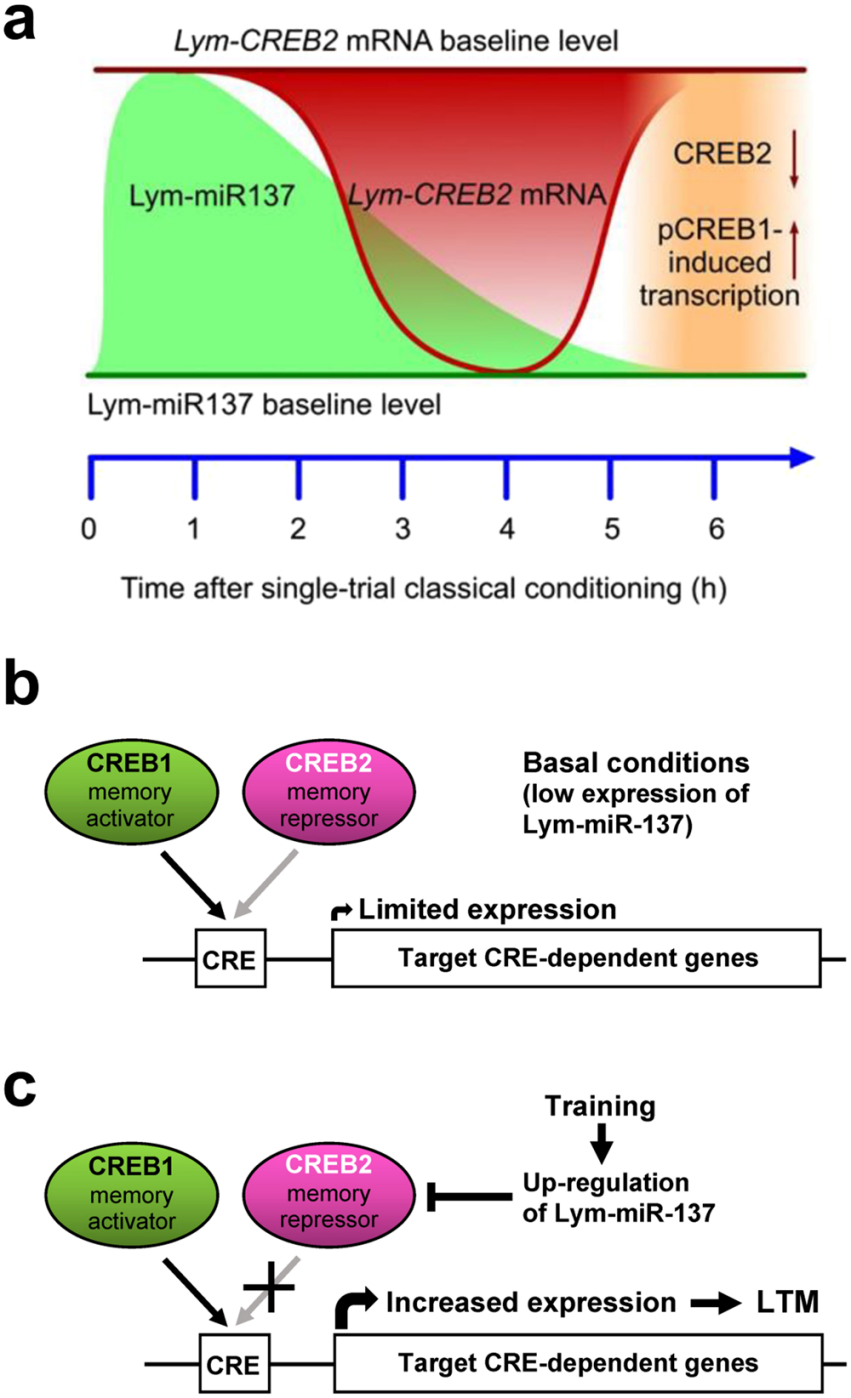
Proposed schematic model for the role of Lym-miR-137 in the regulation of CREB-dependent LTM formation. (**a**) A schematic of the proposed temporal relationships between the learning-induced changes in Lym-miR-137 and *Lym-CREB2* mRNA levels and pCREB1-induced gene transcription. Lym-miR-137 levels were measured at 1 h and 6 h post-training, *Lym-CREB2* mRNA levels were measured at 4 h and 6 h post-training, pCREB1 levels were measured and transcription dependence established at 6 h post-training (see Results for more detail). (**b**) Under basal conditions, CREB1 and CREB2 compete for binding to the cAMP response elements (CRE). Consequently, expression of CRE-dependent genes remains limited. (**c**) Single-trial training increases Lym-miR-137 expression resulting in down-regulation of CREB2. The repressive effect of CREB2 is decreased and CREB1 activates the expression of target CRE-dependent genes required for LTM formation.

Although we consider our findings conclusive with respect to a requirement for Lym-miR-137 in the formation of LTM, they are still open to alternative (albeit not necessarily exclusive) interpretations regarding the time of this requirement. Since we do not know when exactly the mirVANA miRNA inhibitor binds to Lym-miR-137, we cannot rule out the possibility that the inhibitor increases the basal level of CREB2 by blocking Lym-miR-137 before as well as after single-trial training thus affecting the acquisition as well as the consolidation of the 24-h memory. We cannot rule out either that the long-lasting inhibition of Lym-miR-137 by mirVANA miRNA inhibitor and the resulting increase in basal levels of CREB2 could have some effect on future learning in general including single-trial conditioning performed under these conditions. However, the use of the specific miR-137 inhibitor together with the use of PLL and in conjunction with the measured learning-induced increase in Lym-miR-137 and subsequent decrease in *Lym*-CREB2 mRNA levels during the consolidation phase of memory (Fig. 6) lends strong support to the conclusion that in the first few hours after training there is a period of requirement for Lym-miR-137 for single-trial induced LTM.

It is important to note that in addition to CREB2, another member of the ATF/CREB family, CREB1 also regulates long-term synaptic plasticity and memory but it acts as a key transcriptional activator of learning-induced downstream cascades underlying LTM formation^26^. Interestingly, it was shown in the sea slug *Aplysia* that the ratio of CREB1/CREB2 expression can determine the consolidation of LTM and when it is increased, the probability of activating the transcriptional machinery necessary for LTM is higher^22^. Furthermore, previous studies on *Lymnaea* have demonstrated that *CREB1*-specific siRNA injected into the CGCs blocked while *CREB2*-specific siRNA augmented the enhancement of excitatory post-synaptic potentials in the B1 motoneurons, a readout of learning-induced presynaptic facilitation in the CGCs^21^. In other words, preventing the synthesis of new CREB2 molecules by exogenously-produced small non-coding RNAs resulted in reducing CREB2-mediated repression of the effect of CREB1. Our study now reveals an endogenous miRNA-dependent mechanism by which CREB2 may be down-regulated to facilitate transcription-dependent consolidation of LTM around 6 h after single-trial conditioning (Fig. 6). Of note, some of the first evidence that sncRNAs are involved in long-term synaptic plasticity and memory came from experiments conducted on *Aplysia* sensory neurons. In particular, it was shown that miR-124 is an important component of the CREB1-dependent intracellular machinery that converts short-term facilitation into long-term facilitation^6^. Also of significant interest are studies demonstrating that piwi-interacting RNAs (piRNAs) control the methylation state of the *CREB2* promoter and hence enhance memory-related synaptic plasticity^25^. Thus, collectively, recent findings indicate an important role of sncRNA-based mechanisms controlling the CREB1/CREB2 ratio in the formation of new memories. Furthermore, previous work in *Aplysia*^40^ and *Lymnaea*^34^ has indicated that the early stage of memory consolidation is associated with phosphorylation of CREB2 by mitogen-activated protein kinase (MAPK). Since the repressive effect of miRNAs is relatively slow in onset, we believe that Lym-miR-137 is more suited for removing CREB2-mediated transcriptional repression in the later stage of memory consolidation.

MiR-137 has been of great interest due to its role in the regulation of neural development and neoplastic transformation [for a review, see^41^]. An additional important point emerges from recent experiments implicating miR-137 in memory formation in mammals. However, from the limited amount of information available in the literature, there is some controversy concerning the mode of miR-137 action. Indeed, whilst Yi’s group demonstrated that miR-137 is a positive regulator in the formation of spatial memory^17^, Tsai’s group provided evidence that overexpression of miR-137 impairs synaptic plasticity and contextual fear conditioning^18^. Also, taking into account the data we report here, it is tempting to speculate that the observed difference in the role of miR-137 in memory can be explained by differences in the learning paradigms employed. Thus, we can suggest that by interacting with different target RNAs miR-137 initiates different downstream events, depending on the associations used to induce long-lasting memory. It is well-known from the learning and memory literature that the same upstream molecular components can play different roles in different types of learning and the recent miR-137 findings obtained by using three different paradigms fit well into this general framework.

## Material and Methods

### Experimental animals

Specimens of *Lymnaea stagnalis* were raised in the breeding facility of the University of Sussex, where they were kept in 20–22 °C copper free water under 12 h light and dark cycle. They were fed on lettuce 3 times and a vegetable-based fish food twice a week.

### Single-trial conditioning protocol

Reward conditioning was performed using a method based on a previously published protocol^11^. Snails were randomly assigned to experimental (paired) and control (unpaired) groups to be given a single conditioning and control trial, respectively. Experimental animals were exposed to a solution of amyl acetate (CS) and immediately after that to a sucrose solution (US). Control animals were exposed to the CS and to the US, separated by an interval of 1 h. A randomly chosen subset of 20 animals from each group was retained and tested for LTM formation at 24 h after the paired and unpaired trials, as described previously^42^. A third group of animals was kept under the same conditions and had the same feeding regimen as experimental and unpaired control snails but was not exposed to either the CS or the US. This group is referred to as the naïve control group.

### Inhibition of Dicer-mediated biogenesis of miRNAs

Poly-L-Lysine (PLL, Sigma-Aldrich, UK) was used to impair Dicer activity. To examine the potential adverse effects of PLL on the feeding behavior of snails, injection solutions containing different concentrations of PLL (0 µM, 25 µM, 50 µM, 100 µM and 200 µM) were prepared in saline and injected into the haemolymph of snails (n = 12 for each of the four groups). Snails were subjected to feeding tests with sucrose (the US) 2 h prior the injection and 2 h, 4 h, 6 h and 24 h, respectively, after the injection. Based on the results of the test, 25 µM PLL was chosen for the experiments in which we aimed to determine the global functions of miRNAs in long term memory formation through inhibition of Dicer. Three groups of animals were used. Snails from the first group (n = 12) were injected with PLL 2 h before training and snails from the second (n = 12) and the third groups (n = 24) 15 min and 4 h after training respectively. Based on previous measurements of the *in vivo* clearance of intravenously applied FITC-labeled PLL in rats^43^, we estimated that the effect of PLL on Dicer lasted for a maximum of 40 min post-treatment. This means that the effects of the pre-training and post-training impairment of Dicer could be investigated using distinct time-windows. All animals were tested with amyl acetate (the CS) 24 h after training to evaluate long term memory formation.

### miRNA cDNA library construction

Snails were trained using the one-trial conditioning protocol (see above). 1 h and 6 h after training, a randomly chosen subset of animals (n = 10 per time point) was sacrificed and the ‘learning’ ganglia (cerebral and buccal ganglia connected via the cerebral-buccal connective) were dissected from the CNS. The ‘learning’ ganglia were also extracted from animals, which were not exposed to either the CS or US. This group is referred to as naive control. RNA preparations enriched in short non-coding RNA molecules (sncRNA) were isolated from each of the three groups with the help of the miRACLE kit (Stratagene). The purified sncRNAs were used to produce sncRNA cDNA libraries by means of the NEBNext Small RNA Library Prep Set for Illumina according to the manufacturer’s protocol (New England BioLabs). Gel-purified bar-coded sncRNA cDNA libraries were subjected to next-generation sequencing on the Illumina platform at the UCL Genomics (UCL Institute of Child Health, London).

### Next-generation sequencing data analysis

5′ and 3′ adapter sequences were trimmed using Cutadapt^44^ and reads shorter than 15 nt or without adapters were excluded from further analysis. The data were than pre-processed using the Fastx-toolkit. Ribosomal RNAs, ribozymes, transfer RNAs and small nuclear RNAs were also excluded from further analysis. After data pre-processing, the sncRNA reads were aligned against known hairpin sequences deposited in the miRBase database using Bowtie (version 2.2.4). The produced SAM files were post-processed using Python scripts to determine the frequency of each read. The data were than normalised and miRNAs showing learning-induced changes in their expression were identified by the A-C test. Computed p-values were corrected for multiple hypothesis tests by using the Benjamini-Hochberg procedure^45^. miRNAs were considered differentially expressed, if they had a corrected p-value < 0.01.

### Quantitative real-time RT-PCR

RNA preparations enriched with small non-coding RNA molecules (sncRNA) were isolated from the ‘learning ganglia’ dissected from naïve snails and at different time points after training from conditioned and unpaired control snails (n = 10 in each group) by means of the miRACLE kit (Stratagene). The purified RNAs were copied into cDNAs by the TaqMan MicroRNA Reverse Transcription kit (Life Technologies) and the resulted cDNAs were subjected to quantitative PCRs using the TaqMan Universal PCR Master Mix (Life Technologies). Predesigned TaqMan Small RNA Assays 004523 (Life Technologies; target sequence UUAUUGCUUGAGAAUACACGUA) and 000325 (Life Technologies; target sequence UUUUGAUUGUUGCUCAGAAAGC) were used in these quantitative experiments to detect Lym-miR-137 and Lym-miR-315 (endogenous control) respectively. The amount of target Lym-miR-137, normalized to an endogenous reference and relative to a calibrator (CAL), was calculated as 2^−ΔΔCT^, where ΔΔCT = ΔCT − ΔCT(CAL). ΔCT and ΔCT(CAL) are the differences in threshold cycles for the target (Lym-miR-137) and reference (Lym-miR-315) measured in the samples and in the calibrator, respectively^46^.

In order to quantify *Lym-CREB2* mRNA total RNAs were extracted from brain tissue by means of the Absolutely RNA Microprep kit (Agilent Technologies) and treated with DNase TURBO (Ambion). Purified RNAs were copied into cDNAs using the iScript cDNA synthesis kit (Bio-Rad). cDNAs were amplified and analyzed on the Mx3000P real-time cycler (Stratagene) using the FastStart Universal Green Master kit (Roche). We used primers 5′-GTTTGGTGTACAGATGCATAC-3′ and 5′-CTCCAGTTTGGCTGTTCAC-3′ for detection of *Lym-CREB2* mRNA and primers 5′-AAGGGACATTACACAGAGG-3′ and 5′-GTGTCAGTTGGAATCCTTG-3′ for detection of β-tubulin. The amount of *Lym-CREB2* mRNA, normalized to an endogenous reference (β-tubulin mRNA) and relative to a calibrator, was calculated as 2^−ΔΔCT^.

### RNA analysis in isolated identified neurons

The cell bodies of CGCs were identified and individually dissected from the CNS of *Lymnaea* as described previously^9^. Total RNA extracted from the CGCs (n = 10) by means of the Absolutely RNA miRNA kit (Agilent Technologies) was split into separate halves to analyse the expression of Lym-miR-137 and *Lym-CREB2* mRNA.

### Blocking transcription with Actinomycin D

Brain RNA synthesis was reduced using the transcriptional inhibitor actinomycin D (Act D) (Sigma-Aldrich, Poole, UK). Act D was dissolved in the saline buffer to achieve the required concentration of 50 μM. Injections were performed using 1 ml syringes (Becton Dickinson, Madrid, Spain) and 30 gauge precision glide needles (Becton Dickinson, Drogheda, Ireland). 200 μl of Act D were injected into the hemolymph in the snail’s body cavity. As the estimated volume of the hemolymph is ∼1 ml, the estimated final concentration of Act D was one-fifth (10 μM) of the injected concentration. It is known from a previous study^13^ that the time it takes for Act D to block transcription is approximately 1 h. Therefore to achieve transcriptional inhibition at ~1 h and ~6 h post-training Act D was administered 10 min and 5 h, respectively, after single-trial classical conditioning.

### Silencing of Lym-miR-137 *in vivo*

To silence Lym-miR-137 *in vivo* we used the mirVANA miRNA inhibitor ame-miR-137 (Life Technologies). 200 μl of the Invivofectamine 2/miRNA inhibitor complex containing 50 μl of miRNA inhibitor ame-miR-137 (17 μg), 50 μl of complexation buffer and 100 μl of Invivofectamine 2 were prepared according to the manufacturer’s protocol (Life Technologies). The complex was incubated at 50 °C for 30 minutes and then purified using the Float-A-Lyzer G2 dialysis device according to the manufacturer’s protocol (Spectrum Medical Laboratories). The final volume was adjusted to 2.5 ml with the saline buffer. 100 μl of the purified complex were injected into the haemolymph of 20 snails 24 h or 48 h before training. Two control groups of snails (n = 20 per group) were subjected to the same injection protocol. Animals from the first group were injected with the Negative Control Inhibitor (Life Technologies), which is a random sequence anti-miR molecule that has been validated to produce no identifiable effects on known miRNA function. Animals from the second control group were injected with the Invivofectamine 2 only. All animals were tested with the CS 24 h after training to evaluate long term memory formation.

### Data Availability

All real-time RT-PCR data generated or analysed during this study are included in the published article. All behavioral data used for statistical analysis are available on FigShare (https://figshare.com/s/1ea5bbaeace3c8e4ea4c).

## Electronic supplementary material

Supplementary figures
