# Supplementary material for: A CREB2-targeting microRNA is required for long-term memory after single-trial learning
Source: Sci Rep. 2018 Mar 2;8:3950. doi: 10.1038/s41598-018-22278-w (PMC5834643; doi:10.1038/s41598-018-22278-w)
Supplement: Supplementary file 1 — Supplementary figures [file 41598_2018_22278_MOESM1_ESM.pdf]

A CREB2-targeting microRNA is required for long-term memory after single-trial learning

Sergei A Korneev[1*], Dimitris V Vavoulis[2], Souvik Naskar[1], Varvara E Dyakonova[3], Ildikó Kemenes[1], György Kemenes[1]

[1]Sussex Neuroscience, School of Life Sciences, University of Sussex, Brighton BN1 9QG, UK

[2]RDM Nuffield Division of Clinical Laboratory Sciences, University of Oxford, Clifton BS8 1UB, UK

[3]Koltzov Institute of Developmental Biology, Russian Academy of Sciences, Moscow, 119334, Russia

* To whom correspondence should be addressed. Tel: +44 (0)1273 872809; Fax: +44 (0)1273 678535; Email: s.korneev@sussex.ac.uk

Supplementary Figures

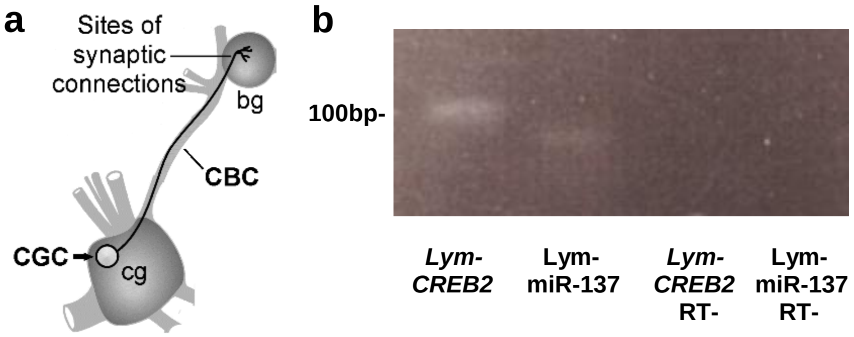

Figure S1. Lym-miR-137 and *Lym-CREB2* mRNA are co-expressed in the serotonergic Cerebral Giant Cell (CGC).

(**a**) The CGC resides within the cerebral ganglion (cg), sends its primary axon to the buccal ganglion (bg) via the cerebral-buccal connective (CBC) where it makes synaptic connections with neurons of the feeding circuit. (**b**) The result of RT-PCR experiment on RNA extracted from isolated CGCs performed with primers specific for the *Lym-CREB2* mRNA and Lym-miR-137. PCR products of the expected sizes were detected. Lanes *Lym-CREB2* RT- and Lym-miR-137 RT- represent control experiments in which reverse transcriptase was omitted. The absence of any PCR products in these lanes proves that the RNA sample used in the RT-PCR experiments was free from DNA contamination. See Fig. S2 for the full-length gel image.

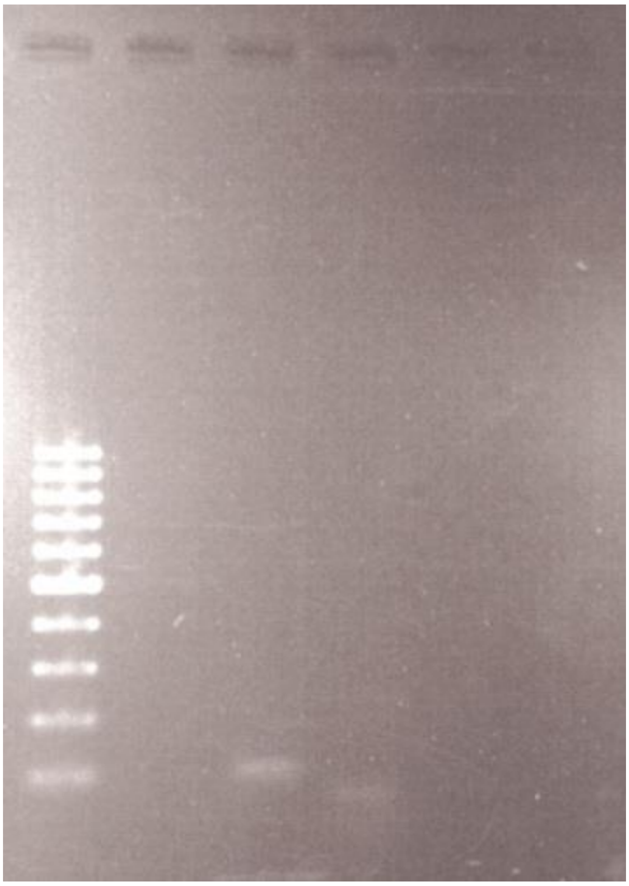

Figure S2. Lym-miR-137 and *Lym-CREB2* mRNA are co-expressed in the CGC.

The full-length gel image showing the result of RT-PCR experiment on RNA extracted from isolated CGCs performed with primers specific for the *Lym-CREB2* mRNA and Lym-miR-137. PCR products of the expected sizes were detected. Lanes *Lym-CREB2* RT- and Lym-miR-137 RT- represent control experiments in which reverse transcriptase was omitted. The absence of any PCR products in these lanes proves that the RNA sample used in the RT-PCR experiments was free from DNA contamination. Lane M – GeneRuler 100 bp DNA ladder (Fermentas).

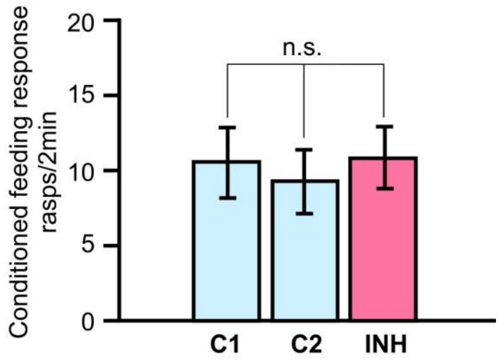

Figure S3. Testing the effect of treatment with the miR-137 inhibitor 24 h before single-trial classical conditioning.

Animals (n = 15 per group) were trained 24 h after injection with either the miR-137 inhibitor ame-miR-137 (INH) or Invivofectamine only (C1) or miRNA inhibitor negative control (C2). All snails were tested for feeding response to the CS (conditioned feeding response) at 24 h after training. Under this condition, there is no impairment of LTM by the miR-137 inhibitor (ANOVA, $p = 0.85$, n.s.). Data are shown as means ± SEM.
